# The Potential Association of CDKN2A and Ki-67 Proteins in View of the Selected Characteristics of Patients with Head and Neck Squamous Cell Carcinoma

**DOI:** 10.3390/cimb46110791

**Published:** 2024-11-20

**Authors:** Dariusz Nałęcz, Agata Świętek, Dorota Hudy, Zofia Złotopolska, Michał Dawidek, Karol Wiczkowski, Joanna Katarzyna Strzelczyk

**Affiliations:** 1 Department of Otolaryngology and Maxillofacial Surgery, St. Vincent De Paul Hospital, 1 Wójta Radtkego St., 81-348 Gdynia, Poland; zzlotopolska@gmail.com; 2 Department of Medical and Molecular Biology, Faculty of Medical Sciences in Zabrze, Medical University of Silesia in Katowice, 19 Jordana St., 41-808 Zabrze, Poland; agata.swietek@sum.edu.pl (A.Ś.); dorota.hudy@sum.edu.pl (D.H.); karolwicz@gmail.com (K.W.); jstrzelczyk@sum.edu.pl (J.K.S.); 3 Silesia LabMed Research and Implementation Centre, Medical University of Silesia in Katowice, 19 Jordana St., 41-808 Zabrze, Poland; 4 Department of Head and Neck Reconstructive Surgery and Robotic Surgery, 1 Powstania Styczniowego St., 81-519 Gdynia, Poland; mdawidek@szpitalepomorskie.eu; 5 Students’ Scientific Association at the Department of Medical and Molecular Biology, Medical University of Silesia in Katowice, 19 Jordana St., 41-808 Zabrze, Poland

**Keywords:** HNSCC, carcinogenesis, protein level, tumor, surgical margin, CDKN2A, Ki-67

## Abstract

Head and neck squamous cell carcinoma (HNSCC) is the sixth most prevalent type of cancer worldwide. Not all mechanisms associated with cell cycle disturbances have been recognized in HNSCC. The aim of this study was to examine the concentration of CDKN2A and Ki-67 proteins in 54 tumor and margin samples of HNSCC and to evaluate their association with the clinical and demographic variables. The ELISA method was used to measure concentrations of CDKN2A and Ki-67 in the tissue homogenates. A significantly higher CDKN2A concentration was found in OSCC tumor samples as compared with OPSCC+HPSCC+LSCC. An inverse correlation was observed for Ki-67. We showed an association between the CDKN2A level and the clinical parameters N in tumors. The patients with concomitant diseases had significantly higher levels of Ki-67 as compared with patients with no concomitant diseases. An analysis of the effect of drinking habits on Ki-67 level demonstrated a statistical difference between regular or occasional users of stimulants and patients who do not use any stimulants in the tumor and margin samples. Moreover, we found an association between CDKN2A and Ki-67 concentrations and the HPV status in tumor and margin samples. The levels of the proteins tested may be dependent on environmental factors. Our results showed that changes in protein levels in HNSCC subtypes may reflect different molecular pathways of tumor development or may also be responsible for the involvement of CDKN2A and Ki-67 in the carcinogenesis process.

## 1. Introduction

Head and neck cancer is the sixth most prevalent type of cancer worldwide where more than 90% of cases represent squamous cell carcinoma [1]. The average age upon diagnosis is 60 years, yet the incidence of such cancers in adults younger than 45 years has increased over the last years due to the higher numbers of oropharyngeal cancers associated with oncogenic human papillomavirus [1,2]. The prognosis for HNSCC prepared by the Polish Ministry of Health projects the growth in incidence by another 10% before 2025 [3]. The main risk factors are HPV infection (especially type 16 and 18), use of tobacco and alcohol abuse, leading particularly to the development of squamous cell carcinoma [1,4]. EBV infection has a role in the development of nasopharyngeal cancers with the greatest carcinogenesis potential upon concomitant infection with HPV [5]. Due to non-specific symptoms, such as xerostomia, dysphagia, gingival bleeding, inflammation of the oral cavity mucous tissue as well as poor social awareness of head and neck cancers, the vast majority of patients are diagnosed at stages III and IV while HNSCC overall survival (OS) is around 1.5 years [6]. A total of 58% of patients with HNSCC show a 5-year survival rate [7]. Better clinical outcomes strongly demand the personalization of treatment as well as risk stratification among patients [8].

Ki-67 is a nuclear antigen showing a role in cell proliferation. The protein is present during the active phases of the cell cycle (G1, S, G2, M) and reaches its peak value during mitosis. During the cell resting phase (G0), no activity of Ki-67 is observed [9]. This protein is considered an important indicator of the cell division rate and used as a diagnostic and prognostic marker in some cancers. Moreover, it is particularly helpful in determination of the tumor’s mitotic activity [10,11,12,13].

The *CDKN2A* (cyclin-dependent kinase inhibitor 2A) gene encodes p16^INK4A^ (p16, CDKN2A) and p14^ARF^ (ARF) proteins, expressed in multiple cell types. The p14^ARF^ protein plays the role of a tumor suppressor, regulating the cell cycle during G1 and G2 phase transition through HDM2 inhibition [14]. The p16^INK4A^ protein takes part in multiple cellular processes, including promotion of proliferation, inhibition of apoptosis and inducing angiogenesis in the cancer cells [15]. Its role is to inhibit the cyclin-dependent kinases (CDK4 and CDK6), thereby activating the retinoblastoma (Rb) family of proteins. This results in suppression of the cell transition from the G1 to the S phase of the cell cycle [14]. Changes in expression of *CDKN2A* gene are associated with the disruption of the normal cell cycle functions, leading to uncontrolled cell proliferation and therefore the implication of neoplastic transformation, so far described in a wide range of cancer types, including melanoma, lymphoma, head and neck squamous cell carcinoma, esophageal squamous cell carcinoma, oral cavity cancer, epithelial ovarian cancer, pancreatic adenocarcinoma, gastric cancer, colorectal cancer, non-small cell lung cancer, prostate cancer and many others [16,17,18,19,20,21,22].

Given the role of CDKN2A and Ki-67 proteins in important cellular processes, including the proliferation and regulation of the cell cycle, it was hypothesized that the levels of those proteins would be changed in the tumor samples as compared with the margin samples depending on selected demographic and clinical–pathological characteristics in our study group with head and neck squamous cell carcinoma (HNSCC). This study aimed at the evaluation of CDKN2A and Ki-67 concentrations and the possible association of the clinical and demographic variables in tumors and the resected surgical margin samples from patients with primary HNSCC.

Based on our knowledge (PubMed, Medline databases), this has been the first study to analyze the concentrations of CDKN2A and Ki-67 proteins in tumor and surgical margin homogenates obtained from patients with HNSCC by immunoassay (ELISA). 

This study aimed at the evaluation of CDKN2A and Ki-67 concentrations and the possible association of the clinical and demographic variables in tumors and the resected surgical margin samples from patients with primary HNSCC.

## 2. Materials and Methods

### 2.1. Study Population

This study comprised 108 samples (54 tumor samples and 54 samples of the corresponding margin) from patients diagnosed with HNSCC. The HNSCC group included 30 cases of oral squamous cell carcinoma (OSCC), 2 cases of oropharyngeal squamous cell carcinoma (OPSCC), 17 cases of laryngeal squamous cell carcinoma (LSCC), 2 cases of hypopharyngeal squamous cell carcinoma (HPSCC), 2 cases of nasal squamous cell carcinoma (NCSCC) and 1 case of skin squamous cell carcinoma (SSCC). The cancer samples and the corresponding margins were collected after surgical resection and were histologically diagnosed by a pathologist. The samples collected were histologically assessed and classified as primary HNSCC. The histologically confirmed cancer-free specimens were taken from the surgical margin at a distance of at least 10 mm from the tumor margin. All the patients were diagnosed, and samples were collected at the Department of Otolaryngology and Maxillary Surgery, St. Vincent De Paul Hospital, Gdynia. The tumor and margin samples were divided into two parts. DNA was first isolated from the samples intended for HPV analysis and then frozen at −80 °C. Samples intended for protein analyses were immediately frozen at −80 °C until further analyses. The study was approved by the Bioethical Committee, Regional Medical Chamber in Gdansk (no. KB-42/21). The laboratory analyses were all carried out in the Department of Medical and Molecular Biology, Faculty of Medical Sciences in Zabrze, Medical University of Silesia. The main inclusion criteria for the HNSCC group included diagnosis of primary squamous cell carcinoma and no preoperative radio- or chemotherapy. All the participants delivered their written informed consent to take part in the study which was also the inclusion criteria. Demographic data of the study group are illustrated in Table 1.

Tumor samples were assessed in accordance with the TNM classification. The tumor stage was categorized according to the 8th edition of the AJCC Cancer Staging Manual [23]. Three patients were staged T1 (5.56%), 10 samples with T2 (18.52%), 19 cases with T3 (35.19%) and 20 patients with T4 (37.04%). Lymph node metastasis was found in 20 patients, i.e., N1 in 6 (11.11%), N2 in 12 subjects (22.22%) and N3 in 2 samples (3.70%). In 15 patients (27.78%) the cancer’s grade was G1, almost 60% of patients (32; 59.26%) were graded G2, 6 subjects (11.11%) were G3 and 1 sample (1.85%) was G4.

### 2.2. Tissue Homogenisation

The tumor and the surgical margin samples were homogenized in cooled PBS buffer (EURx, Gdansk, Poland) at the ratio of 9:1 (PBS volume/tissue weight). Homogenization was conducted using a Bio-Gen PRO200 homogenizer (PRO Scientific Inc., Oxford, CT, USA) at the rate of 10,000 rpm. Subsequently, the homogenates were sonicated using a UP100H ultrasonifier (Hielscher, Teltow, Germany).

### 2.3. Determination of CDKN2A and Ki-67 Protein Concentrations

The enzyme-linked immunosorbent assay (ELISA) was used to determine the concentrations of selected proteins in the homogenates. Commercially available ELISA kits were used for CDKN2A and Ki-67 proteins (SEA794Hu and SEC047Hu, respectively, by Cloud-Clone Corp., Houston, TX, USA). The analyses were performed according to the manufacturer’s instructions. The sensitivity of the detectable CDKN2A dose was 0.262 ng/mL and 0.32 ng/mL for Ki-67. The intra-assay and inter-assay precisions for all kits were <10% and <12%, respectively. All the standards and samples were evaluated in duplicate. The absorbance readings of the samples were recorded at 450 nm with the use of a Synergy H1 microplate reader (Bio-Tek, Winooski, VT, USA). The results were calculated with Gen5 2.06 software (BioTek, Winooski, VT, USA).

### 2.4. Total Protein Concentration Determinations

The quantification of total protein in the homogenates made use of an AccuOrange™ Protein Quantitation Kit (Biotium, Fremont, CA, USA) according to the manufacturer’s protocol. The assay detection range was 0.1–15 μg/mL. Determinations were carried out in duplicate with the previously prepared tissue homogenates, according to the manufacturer’s instructions, without any dilutions. Fluorescence was evaluated with the use of a Synergy H1 microplate reader (BioTek, Winooski, VT, USA) with an excitation wavelength of 480 nm and an emission wavelength of 598 nm. Concentrations of the analyzed proteins were normalized for each sample with reference to the total protein in the tissue lysates, and the values were presented as ng/μg (Ki-67) or pg/μg (CDKN2A).

### 2.5. DNA Isolation and HPV Confirmation

The samples were first homogenized with the use of a Lysing Matrix A (MP Biomedicals, Irvine, CA, USA) and DNA was then isolated using the commercial GeneMATRIX Tissue DNA Purification Kit (Eurx, Gdansk, Poland) according to the manufacturer’s protocol. The quantity and quality of the isolated DNA was assessed using a spectrophotometer NanoPhotometer Pearl (Implen, Munich, Germany). The genetic material was frozen and stored at −20 °C until further analysis.

The presence of HPV was confirmed with a GeneFlowTM HPV Array Test Kit (DiagCor Bioscience Ltd., Kowloon Bay, Hong Kong, China) incorporating the flow-through system FT-PRO (DiagCor Bioscience Ltd., Kowloon Bay, Hong Kong, China) according to the manufacturer’s instructions. The DNA was first used in a PCR reaction on a Mastercycler Personal Thermal Cycler (Eppendorf, Hamburg, Germany). The PCR products were then denatured and hybridized. Following enzyme conjugation and color development, the results were screened using CapturePro Image, CaptureREAD 3.1. (DiagCor Bioscience Ltd., Kowloon Bay, Hong Kong, China). Positive and negative controls were included in all runs.

### 2.6. Statistical Analyses

ELISA results were evaluated with the Shapiro–Wilk test to determine the distribution of the variables. A Student’s *t*-test, a Mann–Whitney *U* test or a Kruskal–Wallis with Dunn–Sidak post hoc were used to verify the significance of differences in the means or medians between the groups. Correlation was established with Spearman’s rank correlation coefficient. The assumed level of significance was *p* < 0.05. The results are presented as mean ± SD or median with quartile range in text. The STATISTICA version 13 software (TIBCO Software Inc., Palo Alto, CA, USA) was used to perform all the analyses. Significant data are shown as box plots with the median in the middle and the 1st and 3rd quartile as a box with minimum and maximum values as whiskers. Data referred to in the text are presented as median with the 1st and 3rd quartile as follows, median (quartile 1st–quartile 3rd).

## 3. Results

### 3.1. Levels of Ki-67 Protein in Tumor and Margin Samples

**Figure 1:**
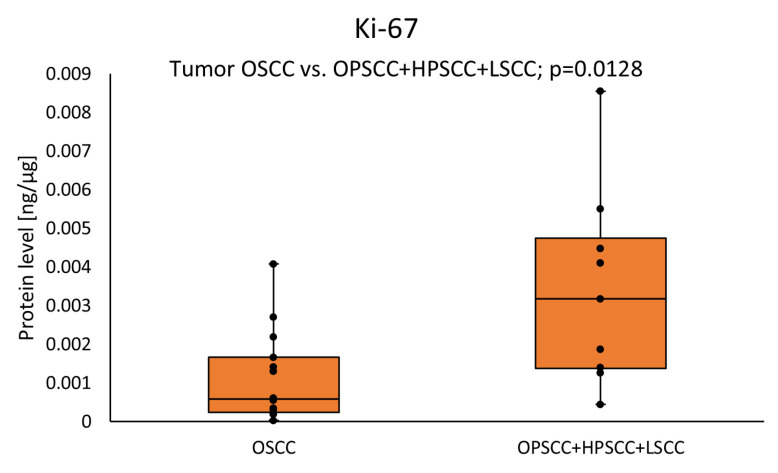
The Ki-67 protein level in the tumor samples according to the tumor subtypes. Statistical analysis with a Mann–Whitney *U* test and differences with *p* < 0.05 are considered statistical.

No significant differences were found in Ki-67 levels in HNSCC tumor samples as compared to the margin samples. A significantly lower concentration of Ki-67 was observed in the OSCC cancer subtype compared with the joint group of OPSCC with HPSCC and LSCC cancer subtypes (0.0005 (0.0002–0.0017) vs. 0.0032 (0.0014–0.0047); *p* = 0.0128) in the tumor samples. The results for the tumor samples are presented in Figure 1.

**Figure 2:**
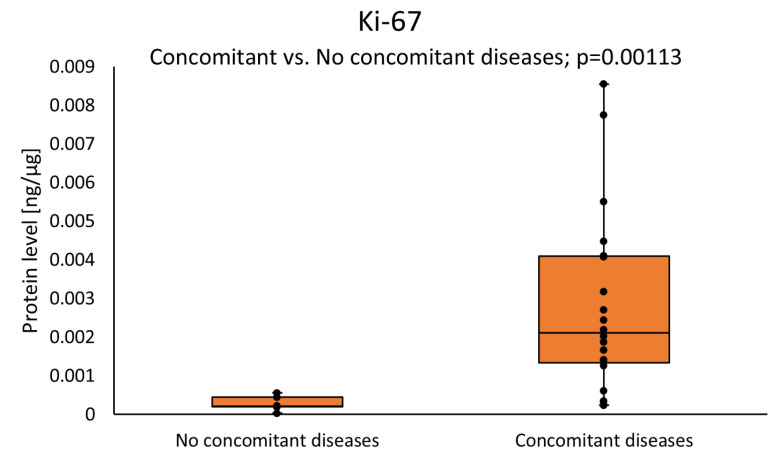
The Ki-67 protein level in the tumor samples according to concomitant diseases status. Statistical analysis was undertaken with a Mann–Whitney *U* test and differences with *p* < 0.05 are considered statistical.

We found that Ki-67 concentration was significantly higher in patients with concomitant diseases as compared with patients without concomitant diseases, as presented in Figure 2 (0.0021 (0.0013–0.0041) vs. 0.0002 (0.0002–0.0004); *p* = 0.0011).

**Figure 3:**
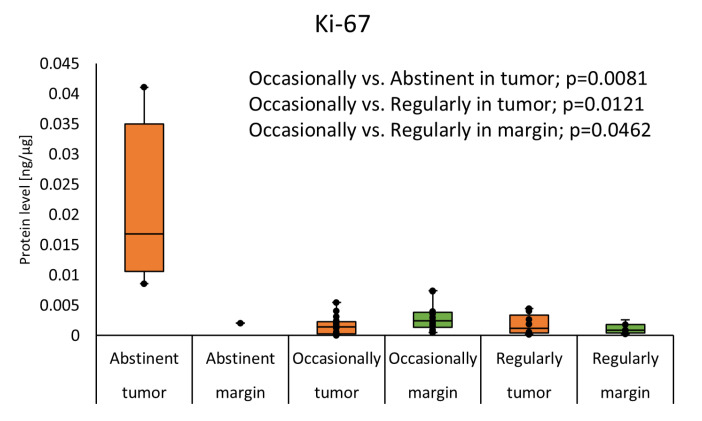
The Ki-67 protein level in tumor and margin samples according to drinking status. Statistical analysis was undertaken with Kruskal–Wallis and Dunn–Sidak post hoc, differences with *p* < 0.05 are considered statistical. The orange color indicates the tumor; green indicates the margin.

The median concentration of Ki-67 was significantly higher in the abstinent individuals as compared with occasional drinkers (0.0169 (0.0106–0.0350) vs. 0.0014 (0.0003–0.0023); *p* = 0.0081), and in abstinents compared with regular drinkers (0.0169 (0.0106–0.0350) vs. 0.0012 (0.0004–0.0034); *p* = 0.0121) in the tumor samples. Moreover, the median concentration of Ki-67 was higher in occasional drinkers compared with regular drinkers (0.0024 (0.0014–0.0039) vs. 0.0009 (0.0004–0.0018); *p* = 0.0462) in the margin. The results are presented in Figure 3.

No other significant differences were found between Ki-67 concentrations and the demographic such as smoking, HPV-DNA and p16 status or clinical–pathological parameters, such as T, N and G classification, except for a positive correlation between Ki-67 protein level in the tumor and the patient’s age (0.59; *p* = 0.0012).

### 3.2. Levels of CDKN2A Protein in Tumor and Margin Samples

**Figure 4:**
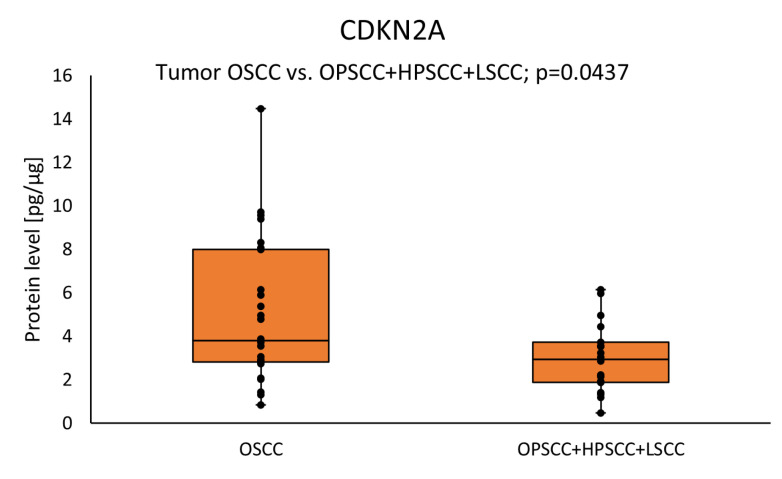
The CDKN2A protein level in the tumor samples according to tumor subtypes. Statistical analysis was undertaken with a Mann–Whitney *U* test and differences of *p* < 0.05 are considered statistical.

No significant differences were found in the CDKN2A level in HNSCC tumors as compared to the margin samples. We observed a significant difference between OSCC and OPSCC with HPSCC and LSCC tumor groups in CDKN2A level (3.8029 (2.8172–7.9817) vs. 2.9439 (1.8819–3.7261); *p* = 0.0437). The CDKN2A level was higher in the OSCC group compared with the joint group of OPSCC, HPSCC and LSCC. The results are given in Figure 4.

**Figure 5:**
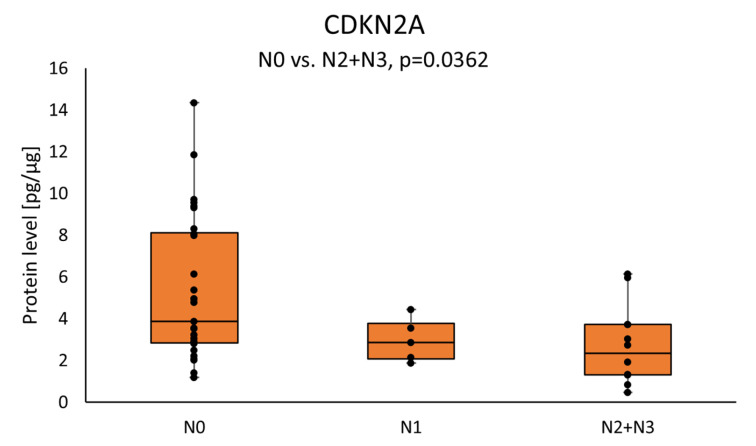
The CDKN2A protein level in the tumor samples according to the patient’s nodal status. Statistical analysis was undertaken with Kruskal–Wallis and Dunn–Sidak post hoc, and differences of *p* < 0.05 are considered statistical.

We observed higher levels of CDKN2A protein in the tumor samples of patients with lower nodal status, N0 vs. N2+N3 (3.8763 (2.8281–8.1203) vs. 2.3377 (1.3179–3.7278); *p* = 0.0362). The results are presented in Figure 5.

**Figure 6:**
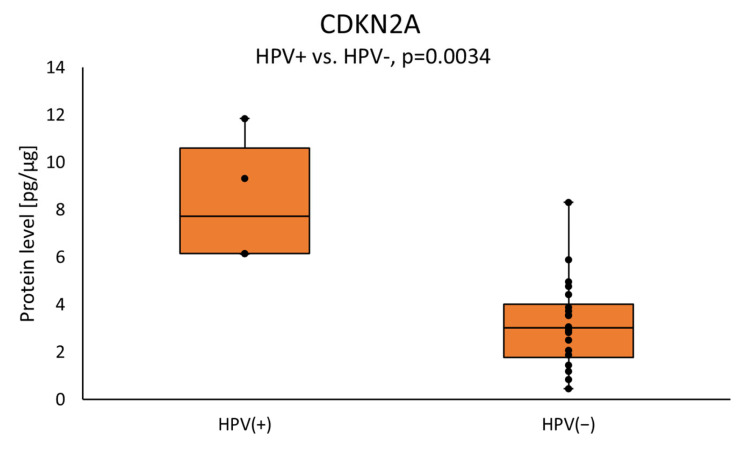
The CDKN2A protein level in the tumor samples according to HPV status. Statistical analysis was undertaken with a Mann–Whitney *U* test and differences of *p* < 0.05 are considered statistical.

The tumor samples from HPV-positive patients showed a higher level of CDKN2A as compared with those with negative HPV status (7.7355 (6.1427–10.592) vs. 3.0240 (1.7708–4.0156); *p* = 0.0034) (Figure 6).

No significant differences in CDKN2A concentration were observed in the margin samples in each of the combinations mentioned above and no other significant differences were found for the demographic or clinical–pathological parameters, such as T and G classification. However, a positive correlation was observed between the CDKN2A protein level in the tumor samples and the smoking years (0.29; *p* = 0.0376), while the margin samples showed a correlation with the number of cigarettes smoked per day (0.30; *p* = 0.0363) and a medium positive correlation with pack-years (0.31; *p* = 0.0367).

## 4. Discussion

CDKN2A and Ki-67 are some of the key proteins associated with regulation of the cell cycle [24,25,26]. However, the exact role of CDKN2A and Ki-67 in prognosis and biological function has not been yet well established in the HNSCC subtypes.

Our analysis showed no statistical differences in the expression levels of CDKN2A and Ki-67 proteins in the tumor samples as compared to the surgical margin samples. However, significantly higher levels of CDKN2A protein were observed in patients with OSCC compared with OPSCC+HPSCC+LSCC. Previous studies have shown that alterations in CDKN2A expression are more frequent in OSCC than in other solid tumors such as pancreatic tumors, bladder cancer, renal cell carcinoma, non-small cell lung cancer, melanoma, glioma, gastroesophageal junction and gastric adenocarcinomas [27,28,29]. This is mainly due to mutations, loss of heterozygosity and DNA hypermethylation of the CDKN2A gene. It is assumed that epigenetic changes in CDKN2A have been linked to genetic or epigenetic changes in other cancer-related genes [14,30]. Some studies have reported increased CDKN2A expression in HNSCC [31,32], while others report decreased CDKN2A expression [33,34,35]. These differences may be due to the type of the material used, including HNSCC tumor tissues, tissues from animal models and the detection method applied, including immunohistochemistry, reverse phase protein array (RPPA) and fluorescence in situ hybridization. Moreover, we can speculate that discrepancies in CDKN2A protein expression may result from the changes in transcription and translation processes and may also be due to tumor heterogeneity [36]. On the other hand, our data show a statistically significant difference in higher Ki-67 levels in the OPSCC+HPSCC+LSCC group as compared with OSCC. This variation in Ki-67 expression reflects the underlying molecular pathways, clinical courses, treatment modalities and outcome differences among the HNSCC subtypes. The study by Ahmed et al. revealed reduced Ki-67 expression in laryngeal tumors compared with oral and pharynx squamous cell carcinomas. Presumably, their results differed from ours because their team studied the genes and used qPCR techniques [37]. Other studies showed abnormal DNA methylation, with hypermethylation occurring predominantly in OSCC cases, while hypomethylation was observed more frequently in LSCC and OPSCC, which may partly explain the differential behavior of HNSCC subtypes, including the altered cell cycle and proliferation [38,39,40].

Moreover, we reported the increased concentration of Ki-67 in patients with concomitant diseases (including cardiovascular diseases, kidney diseases, gastrointestinal diseases, endocrine diseases and others), compared to patients without concomitant diseases. Increased Ki-67 levels have been found in some diseases. such as diabetes, atherosclerosis, rheumatoid arthritis and pancreatitis [41]. A study of oral cancer in rats showed higher expression of Ki-67 in diabetic animals as compared with healthy rats [33]. Other studies have shown that inflammation and/or cancer can affect changes in Ki-67 expression and the cell cycle [41]. We could speculate that higher Ki-67 levels in the group with concomitant diseases may have resulted from a local tissue response to the accompanying inflammation or compensation for the cell damage or death.

Our study demonstrated that the median concentration of Ki-67 in the tumor samples was significantly higher in abstinent individuals as compared with occasional drinkers as well as regular drinkers. Furthermore, the median level of Ki-67 in the margin samples was higher in occasional drinkers compared with regular drinkers. Alcohol consumption is a well-known risk factor for head and neck squamous cell carcinoma, however the underlying molecular mechanisms remain unclear. It has been suggested that ethanol may induce basal cell proliferation, which can consequently cause DNA damage and lead to cancer [42]. Studies of tongue carcinoma, esophageal squamous cell carcinoma and head and neck squamous cell carcinoma cell lines have shown that alcohol inhibits cell proliferation and could have an effect on cell cycle inhibition in the G2/M phase [43,44]. On the other hand, Liu et al. observed increased proliferation in oroesophageal squamous cell carcinoma cell lines exposed to ethanol [45]. The disparate results are possibly dependent on the alcohol dose used and the type of cells. Moreover, it seems that acetaldehyde, which is the first metabolite of ethanol, is able to modify the processes of methylation, DNA synthesis and repair, and may interact with proteins and thus influence the proliferation processes [46,47].

In our study, we observed a positive correlation between Ki-67 levels and age. To date, few studies have described a correlation between Ki-67 expression and age in patients with HNSCC. It has been shown that older patients may have different patterns of inflammation, immune surveillance and cellular aging, which, among other factors, may influence the tumor growth [48]. A study by Jing et al. demonstrated that Ki-67 expression was significantly connected with patient age, presenting that people under 60 years exhibited lower Ki-67 expression [49]. In another recent study Wang et al. showed no statistically significant variance in the mean Ki-67 expression between patients with HNSCC aged 60 or younger and those older than 60 [50]. Similar results were obtained in another histological and immunohistochemical study in OSCC, where no differences in proliferative activity were noted between younger and older patients [51].

Our study reported that CDKN2A protein concentration in the tumor samples in the group of patients with the nodal status N2 or N3 was significantly lower as compared with samples in the group of patients with the nodal status N0. Interestingly, CDKN2A is also occasionally used preoperatively during diagnostic biopsy to predict cancer aggressiveness [52]. The literature available reports that decreased CDKN2A expression is associated with poor prognosis, higher stage and progression of HNSCC [53,54,55,56]. Another study has reported that decreased CDKN2A/p16 expression was associated with OSCC progression and lymph node metastasis [57]. In a Chinese study, the authors reported that methylation, and therefore decreased CDKN2A expression, were associated with the lymph node metastasis in squamous cell carcinomas of the buccal mucosa [58]. In contrast, another team found no significance between CDKN2A methylation and clinicopathological data in OSCC [59]. Interestingly, other studies have confirmed that CDKN2A methylation might be associated with metastasis in other cancer types, including gastric cancer, breast cancer and endometrial cancer [60,61,62].

In our study, CDKN2A protein levels in the study group were higher in HPV-positive DNA tumor tissue. This correlation is attributed to the influence of the E7 oncogene of HPV, which can bind to the tumor suppressor protein pRb and form a complex, which may result in increased levels of CDKN2A. Previous reviews estimated that 22–26% of HNSCCs were HPV-positive [63]. Some studies have demonstrated the utility of p16 as a surrogate marker of HPV infection in HNSCC while other studies have found no such association [64,65]. This suggests the existence of other HPV-independent mechanisms that may affect CDKN2A overexpression, which has been confirmed in cervical cancer and cancers of the oral cavity and pharynx. HNSCC is a heterogeneous disease entity due to its HPV infection status. Therefore, we suggest that there are differences in the oncogenic pathways, probably in addition to viral oncoproteins, which are associated with HPV genes involved in different processes, able to affect multiple molecular pathways.

Furthermore, we reported a positive correlation between the CDKN2A protein level in the tumor samples and years of smoking, while in the marginal samples we observed a positive correlation with the number of cigarettes smoked per day and an average positive correlation with pack-years. As is well known, smoking is one of the best-studied non-genetic risk factors for cancer, which can cause changes in the gene expression profile leading to malignant transformation [66]. The results of most studies have shown that CDKN2A levels can be decreased under the influence of smoking due to changes in the form of deletion or methylation of the gene promoter [18,67,68,69,70,71]. Discrepancies compared with the results of our study may be due to factors such as differences in sampling techniques, preparation and detection methods. Based on our results, the effect of cigarette smoke components may be through modulation of CDKN2A expression in response to epithelial damage. We suspect that alternative genetic and epigenetic mechanisms may exist in smokers that may be involved in altered gene and protein expression, including CDKN2A.

To summarize, the small sample size was the main limitation of this study. Larger and more diverse cohorts should be used to validate our results for the use of these parameters in the monitoring of the clinical course of the disease. Furthermore, such an analysis should include the cell lines to better understand the role these proteins play in head and neck squamous cell carcinoma.

Along with the continuous development of medicine, molecular diagnostics is beginning to play an increasingly important role in oncology. Importantly, the latest ESMO treatment guidelines recognize the role of molecular diagnostics [72]. This underscores the need for further research to help develop available diagnostic and prognostic markers. In the future, a better understanding of the molecular biology of HNSCC should lead to personalized treatment based on genomic alterations or gene expression profiles. This will further enable acceleration and simplification of the diagnostic pathway, reducing the overall time to treatment from the time of cancer diagnosis. In addition, molecular biology analyses can also be used as predictors of possible response to chemotherapy [73].

To the best of our knowledge based on PubMed and Medline databases, this is the first study to have analyzed the concentrations of CDKN2A and Ki-67 proteins in homogenates of tumors and matched surgical margins from patients with HNSCC using an immunoassay (ELISA). In earlier studies, the authors focused on examining the methylation pattern of the CDKN2A gene using methylation-specific PCR and analyzing CDKN2A mutations using polysomic and genomic data, as well as p16 immunoexpression analysis [74,75,76,77,78,79].

## 5. Conclusions

Changes in the protein levels in HNSCC subtypes may reflect the diversity of molecular pathways of tumor development depending on the subtype or may also be responsible for the involvement of CDKN2A and Ki-67 in the carcinogenesis process. The levels of the proteins tested may be dependent also on environmental factors such as alcohol consumption, smoking and HPV status. The results underscore the necessity for a more personalized approach in the treatment of HNSCC, considering the specific molecular and environmental influences on the tumor biology. Future research should comprise larger cohorts and assume long-term follow-up to validate Ki-67 and CDKN2A as reliable prognostic biomarkers and to explore targeted interventions based on these molecular insights.

## Figures and Tables

**Table 1:** Demographic data of the study group.

	N	%
Average age (range)	64 (53–72)	
Female	18	33.33
Male	36	66.67
Smokers	45	83.33
Non-smokers	9	16.67
Alcohol users (occasional)	36	66.67
Alcohol users (regular)	15	27.78
Alcohol non-users	3	5.56

## Data Availability

The data used to support the findings of this research are available upon request.

## Abbreviations

HNSCC	Head and neck squamous cell carcinoma
HPV	Human papillomavirus
CDKN2A/p16	Cyclin-dependent kinase inhibitor 2A
Rb/pRb	Retinoblastoma protein
CDK4	Cyclin-dependent kinase
CDK6	Cyclin-dependent kinase
OSCC	Oral squamous cell carcinoma
LSCC	Laryngeal squamous cell carcinoma
OPSCC	Oropharyngeal squamous cell carcinoma
NCSCC	Nasal squamous cell carcinoma
SSCC	Squamous cell carcinoma
HPSCC	Hypopharyngeal squamous cell carcinoma
AJCC	American Joint Committee on Cancer
PCR	Polymerase chain reaction
ELISA	Enzyme-linked immunosorbent assay
RPPA	Reverse phase protein array
qPCR	Quantitative polymerase chain reaction
